# A benchmark for environmental microorganism object detection on EMDS-7

**DOI:** 10.3389/fmicb.2026.1821911

**Published:** 2026-04-13

**Authors:** Jia Guo, Juanjuan Guo, Bin Yan

**Affiliations:** 1 School of Mathematics and Statistics, Hunan First Normal University, Changsha, China; 2 The Second Clinical Hospital of Lanzhou University, Lanzhou, China

**Keywords:** benchmark, EMDS-7, environmental microorganisms, microscopy, object detection

## Abstract

Environmental microorganism (EM) detection from microscopy images supports scalable water-environment monitoring, but fair comparison of detection algorithms is hindered by inconsistent experimental protocols and limited benchmark reporting. We construct a reproducible EM object detection benchmark on the EMDS-7 dataset and, using a fixed split, unified input normalization, and from-scratch training without external pretraining, evaluate 25 representative detectors spanning two-stage proposal-based methods, one-stage dense detectors, keypoint-based formulations, and Transformer-based end-to-end approaches. Performance is measured with COCO-style mAP over IoU 0.50:0.05:0.95, complemented by AP across IoU thresholds, recall-based analysis, and backbone comparisons across ResNet-18/50/101 where supported. Two-stage detectors achieve the strongest overall accuracy on EMDS-7, with Faster R-CNN obtaining the best mAP (64.0%) and Cascade R-CNN close behind (63.9%), while modern one-stage detectors substantially narrow the gap, exemplified by RTMDet-X (60.9%). AP consistently decreases as IoU becomes stricter, indicating that precise localization is a key bottleneck in EM microscopy; backbone scaling does not yield uniform gains under from-scratch training, and recall analysis reveals distinct operating characteristics across paradigms, including high-recall but lower-precision tendencies for some methods. These results suggest that EMDS-7 favors detectors with robust localization and controlled false positives under cluttered microscopic backgrounds, and that future progress should emphasize high-IoU localization for small instances, boundary-aware learning, and false-positive suppression rather than relying solely on deeper backbones; the proposed benchmark provides reproducible baselines and diagnostic evidence to guide subsequent EM detection research.

## 1 Introduction

Environmental microorganisms (EMs) constitute a fundamental component of aquatic ecosystems, and variations in their abundance and composition can serve as sensitive indicators of water quality and ecological conditions. In operational monitoring of urban and peri-urban waters (e.g., lakes and rivers), microscopy remains a primary modality for observing microorganisms. Consequently, developing computational methods that can

automatically *localize* and *identify* multiple microorganism instances in microscopy images is of substantial interest for enabling scalable, objective, and reproducible assessment workflows.

Traditional EM analysis pipelines typically depend on laboratory-based assays and manual microscopic inspection performed by trained experts. While these approaches can be reliable, they often entail high labor cost, limited throughput, and notable inter-operator variability. Moreover, environmental microscopy images exhibit several domain-specific challenges: microorganisms frequently appear as small objects; morphological diversity leads to large intra-class variation; visually similar taxa introduce high inter-class similarity; and complex backgrounds with debris, impurities, and uneven illumination can induce spurious detections. These characteristics make EM detection markedly different from generic natural-image detection, motivating dedicated benchmarking and methodological development.

In recent years, deep learning (DL) has reshaped computer vision, and object detection (OD)—the task of predicting both the category label and the spatial extent (e.g., bounding boxes) of each object instance—has achieved impressive progress on large-scale generic benchmarks. However, transferring OD models to EM microscopy is non-trivial. A central bottleneck is the limited availability of publicly accessible datasets with standardized instance-level annotations and well-defined evaluation protocols tailored to multi-object environmental microscopy. Without a common benchmark, comparisons across studies are confounded by discrepancies in dataset splits, annotation conventions, and metric choices, which hinders reproducibility and slows scientific progress.

To address this gap, the Environmental Microorganism Image Dataset Seventh Version (EMDS-7) was introduced as a dataset explicitly curated for multi-object detection in environmental microscopy. EMDS-7 provides microscopy images spanning dozens of microorganism categories with instance-level bounding-box annotations in a widely used format, allowing direct adoption by mainstream OD frameworks. In addition, EMDS-7 includes a unified label for unknown targets and background artifacts to better approximate real-world monitoring conditions, where open-set observations and non-biological noise are common.

Building upon EMDS-7, this paper establishes a benchmark for environmental microorganism object detection. We define a reproducible training and evaluation protocol and conduct systematic comparisons among representative OD architectures under controlled settings. Beyond reporting aggregate performance, we perform error analyses that emphasize domain-relevant failure modes, including localization errors on small instances, confusions among visually similar categories, performance degradation under long-tailed class distributions, and false positives induced by cluttered backgrounds. The resulting baselines and diagnostics aim to provide a dependable reference for subsequent algorithmic improvements and to inform the design of OD systems for practical water-environment monitoring applications.

## 2 Related work

### 2.1 Environmental microorganism datasets

Environmental microorganism microscopy datasets are a key prerequisite for data-driven water-environment monitoring and automated analysis (Kulwa et al., 2019; Ma et al., 2023). Beyond enabling model training, well-curated public datasets with consistent annotation rules and reproducible splits are essential for fair comparison across methods and for reliable ablation studies.

Environmental microorganism image analysis commonly involves classification, segmentation, and object detection (Zhao et al., 2022a; Ma et al., 2023). Among them, object detection (OD) directly supports practical workflows that require instance-level localization and counting, since a single microscopy field of view often contains multiple co-occurring taxa with highly imbalanced frequencies and substantial background clutter (Zhong et al., 2025).

Constructing such datasets is non-trivial (Li et al., 2021). Variations in acquisition conditions (e.g., magnification and illumination) can cause domain shift across sources (Bhattacharya et al., 2025). Moreover, microorganisms exhibit fine-grained morphology, leading to large intra-class variation and high inter-class similarity. In addition, targets are frequently small and densely distributed, making instance-level annotation expensive and prone to missed labels and inconsistent bounding (Gogoberidze and Cimini, 2024). Long-tailed class distributions further complicate evaluation by increasing variance for rare categories (Liu X. et al., 2022). To improve reproducibility in this domain, recent benchmark-oriented efforts have provided more standardized taxonomies, instance-level annotations, and fixed protocols (Zhao et al., 2022a). The EMDS series represents one such line of work, and EMDS-7 in particular is organized for multi-object detection with instance-level annotations and an additional label to account for unknown targets and artifacts, helping approximate real-world microscopy observations with incomplete label spaces and non-biological noise (Yang et al., 2023).

### 2.2 Object detection techniques

Object detection (OD) aims to localize and classify each object instance in an input image, typically producing bounding boxes with confidence scores. In modern deep learning pipelines, an OD system is usually composed of (i) a backbone network for feature extraction (He et al., 2016), (ii) a feature aggregation module (often multi-scale) (Lin et al., 2017a), and (iii) prediction heads for classification and bounding-box regression. Localization quality is commonly measured by Intersection over Union (IoU) (Rezatofighi et al., 2019), while duplicate predictions at inference time are usually removed via post-processing such as Non-Maximum Suppression (NMS) (Hosang et al., 2017).

From an architectural perspective, mainstream detectors are often grouped into two-stage and one-stage paradigms. Two-stage methods first generate candidate regions (region proposals) and then perform classification and refined regression on these proposals. Representative frameworks rely on a Region Proposal Network (RPN) (Ren et al., 2016) and Region of Interest (RoI) feature alignment (e.g., RoI pooling/alignment) (Girshick, 2015; He et al., 2017). They often achieve strong localization accuracy and competitive recall, but typically incur higher computational cost. In contrast, one-stage detectors perform dense predictions directly on feature maps (with or without anchors) (Redmon et al., 2016; Liu et al., 2016), offering simpler pipelines and higher inference efficiency. However, one-stage training is more susceptible to class imbalance between foreground and background and to the dominance of easy negatives; accordingly, many approaches adopt tailored loss functions (Lin et al., 2017b) and assignment strategies (Zhang et al., 2020) to stabilize optimization and improve learning on hard examples.

More recently, Transformer-based detectors have emerged as an additional paradigm. A representative idea is to formulate detection as set prediction, where a fixed-size set of predictions is matched to ground-truth instances through bipartite matching (Carion et al., 2020). This design reduces reliance on hand-crafted anchor configurations and can lessen the need for complex heuristic post-processing. Transformer-based detectors benefit from global context modeling and end-to-end training, but practical performance for high-resolution inputs and small objects often depends on incorporating multi-scale features and more efficient attention mechanisms (Zhu et al., 2020).

Beyond model families, training and implementation choices can substantially affect OD performance and reproducibility (Zhang Z. et al., 2019; Wang et al., 2023). Input resolution and multi-scale training influence the visibility of small instances; data augmentation affects robustness to imaging variations; loss design and sample assignment determine the optimization emphasis across classification and localization; and inference-time thresholds together with NMS settings govern the trade-off between recall and false positives. Therefore, when establishing a benchmark, it is critical to control these factors under a unified protocol (e.g., fixed splits, training schedules, input scales, and post-processing rules) to ensure fair and interpretable comparisons across detection frameworks (Singh et al., 2024).

### 2.3 Object detection for environmental microorganisms

Applying generic object detection models to environmental microorganism microscopy introduces pronounced domain-specific difficulties. First, microorganisms often appear as small instances with weak texture, blurred boundaries, and low contrast; crowded fields of view may further amplify localization errors due to dense layouts and partial overlaps (Sun et al., 2025). Second, environmental samples typically contain substantial non-biological interference (e.g., debris, bubbles, and particulate impurities) and are affected by imaging variability such as non-uniform illumination, sensor noise, and slide-preparation differences. These factors can substantially increase false positives and reduce stability across sampling conditions (Khodaparast et al., 2017; Ahmad et al., 2017; Zhang et al., 2026). Third, microorganism categories are frequently fine-grained: the same taxon can exhibit large appearance changes across growth stages, poses, and aggregation states, while different taxa may be visually similar, making category confusion a common failure mode (Chin et al., 2024; Nishimura et al., 2025). Finally, real-world sampling often yields imbalanced, long-tailed class frequencies, which biases detectors toward frequent classes and leads to volatile performance on rare categories (Hou et al., 2021; Li et al., 2026).

To address these challenges, existing studies typically adapt and strengthen generic detectors using several recurring strategies. One line of work focuses on small-object sensitivity by increasing input resolution, adopting multi-scale training and testing, and using feature pyramid designs to combine low-level details with high-level semantics (Cai et al., 2025; Wu et al., 2022). A second line targets robustness to clutter and artifacts through domain-relevant augmentation (e.g., photometric perturbations and noise simulation) and through attention mechanisms or contextual modeling that encourage the network to focus on informative regions while suppressing background responses (Zhao et al., 2022b). A third line aims to reduce fine-grained confusions and class-imbalance effects via more robust classification objectives, re-weighting schemes, improved sample assignment, and hard example mining; some approaches additionally employ class-balanced sampling or prior-guided constraints during optimization (Wen et al., 2024). A fourth direction concerns dense-scene post-processing: when instances are close to each other, standard Non-Maximum Suppression (NMS) may over-suppress true positives, so tuning thresholds or adopting softer suppression variants can be beneficial for recall.

Despite encouraging progress, a fundamental obstacle remains for this domain: results are often difficult to compare across studies due to discrepancies in datasets, label taxonomies, annotation conventions, data splits, and evaluation settings. This issue is especially pronounced when small objects and long-tailed distributions coexist, because performance can be highly sensitive to implementation choices such as input scale, training schedules, augmentation policies, and inference-time thresholds. Without a unified protocol, it is hard to attribute performance differences to model design rather than experimental configuration (Yang et al., 2023).

Consequently, establishing a standardized benchmark is particularly valuable for environmental microorganism object detection. By fixing data splits, training configurations, and evaluation metrics, and by comparing representative detector families under controlled conditions, a benchmark can provide reproducible baselines and reveal domain-relevant error modes, such as small-instance localization failures, confusions among visually similar taxa, and false positives triggered by complex backgrounds. These diagnostics can in turn guide more targeted model and training improvements toward reliable, deployable detection systems for water-environment monitoring.

## 3 Benchmark setting

This chapter presents the benchmark protocol for environmental microorganism object detection on EMDS-7. We specify the dataset and split policy, the unified training and input processing procedure, and the evaluation metrics with formal definitions, so that different detectors can be compared under consistent and reproducible conditions and the observed performance differences can be attributed primarily to model design.

### 3.1 Dataset and split protocol

The benchmark follows a Pascal Visual Object Classes (Pascal VOC) style instance annotation scheme: each microscopy image is associated with an annotation file that records the category label and bounding box for every instance. Images are stored in PNG format. The dataset contains 42 categories (including the *unknown* category) with approximately 2,365 images in total. To ensure stable and reproducible comparisons, we adopt a pre-defined and fixed train/validation split rather than random re-splitting; the split ratio is approximately 8:2 in terms of the number of images. Using a fixed split reduces variance introduced by random partitioning and mitigates potential data leakage, thereby improving the interpretability of cross-model comparisons. Beyond the basic dataset statistics, EMDS-7 presents several properties that make it particularly challenging for object detection. Many microorganism instances are small relative to the image size, so slight coordinate deviations may lead to substantial IoU degradation. In addition, object boundaries are often ambiguous under microscopy, and different categories may exhibit considerable visual similarity despite belonging to distinct taxa. The dataset also contains cluttered backgrounds, including impurities, debris, and other non-biological interference, which can increase false positives and complicate localization. These characteristics make EMDS-7 a challenging benchmark not only for classification but also for accurate instance localization and robust false-positive suppression.

An additional characteristic of EMDS-7 is the inclusion of an unknown category. This category is intended to capture targets that cannot be reliably assigned to the predefined microorganism taxonomy, as well as certain visually confusing structures or artifacts encountered in realistic microscopic observations. By including this category, the dataset better reflects practical monitoring scenarios in which not all observed instances belong to a closed and perfectly annotated label space. At the benchmark level, the unknown category increases the realism and difficulty of the task, since detectors must distinguish annotated microorganism classes not only from each other, but also from ambiguous or hard-to-classify instances.

### 3.2 Unified training and input processing

To enable fair comparison, all detectors are trained under a unified protocol that preserves only method-essential differences. Specifically, all models are trained from scratch without external pre-trained weights, and sources of randomness are controlled to enhance reproducibility. For input processing, we apply a consistent geometric normalization strategy for both training and validation: images are resized while preserving the aspect ratio with a standard detection constraint (maximum side 1, 333 and minimum side 800). The training pipeline follows the order: image loading, instance annotation loading (bounding boxes), aspect-ratio-preserving resizing to (1, 333, 800), random horizontal flipping with probability 0.5, and input packaging for the detector. The validation pipeline is deterministic and follows the order: image loading, aspect-ratio-preserving resizing to (1, 333, 800), annotation loading, and input packaging, without any random augmentation. These settings aim to reduce confounding effects from input scale, augmentation, and training procedure, ensuring that performance differences mainly reflect detector architectures and learning mechanisms.

While the unified protocol is intended to improve fairness and reproducibility, we acknowledge that different detectors may exhibit substantially different optimization preferences and convergence behaviors. As a result, a fully standardized setting may not always allow each method to reach its own best-case performance. In this benchmark, our objective is not to reproduce the individually optimized results reported in the original literature, but to provide a controlled comparison on EMDS-7 under consistent data processing and evaluation conditions. For this reason, we unify the split protocol, input normalization, training duration, and randomness control, while preserving a limited degree of method compatibility for essential optimization components when necessary. Accordingly, the reported results should be interpreted as benchmark comparisons under a reproducible common protocol, rather than as the absolute performance ceiling of each detector.

### 3.3 Evaluation metrics

Evaluation is performed on the validation set. We primarily report COCO-style Average Precision (AP) and mean Average Precision (mAP) under multiple Intersection over Union (IoU) thresholds, and additionally report Average Recall (AR) as a complementary measure.

Given a predicted bounding box *B*_*p*_ and a ground-truth box *B*_*g*_, IoU is defined as


$$\text{IoU}\left(B_{p},B_{g}\right)=\frac{\left|B_{p}\cap B_{g}\right|}{\left|B_{p}\cup B_{g}\right|}.$$

For a specified IoU threshold *t*, predictions are ranked by confidence and matched to ground-truth instances to determine True Positives (TP), False Positives (FP), and False Negatives (FN). Precision and Recall are computed as


$$\text{Precision}\left(t\right)=\frac{TP\left(t\right)}{TP\left(t\right)+FP\left(t\right)},\text{ Recall}\left(t\right)=\frac{TP\left(t\right)}{TP\left(t\right)+FN\left(t\right)}.$$

For category *c* at threshold *t*, the Average Precision *AP*_*c, t*_ is defined as the area under the Precision–Recall (PR) curve:


$$AP_{c,t}=\int_{0}^{1}P_{c,t}\left(R\right)dR,$$

where *P*_*c, t*_(*R*) denotes precision at recall *R* (approximated numerically from discrete PR points in practice). Averaging over all *C* categories yields


$$mAP_{t}=\frac{1}{C}\overset{C}{\underset{c=1}{\sum }}AP_{c,t},$$

where *C* = 42 in this benchmark (including *unknown*). We adopt the multi-threshold set *T* = {0.50, 0.55, …, 0.95} and define the overall COCO-style metric as


$$mAP_{0.50:0.05:0.95}=\frac{1}{\left|T\right|}\underset{t\in T}{\sum }mAP_{t}.$$

Accordingly, in Table 1 we report AP50≜*mAP*_0.50_, AP75≜*mAP*_0.75_, and the overall *mAP*≜*mAP*_0.50:0.05:0.95_.

**Table 1:** Best configuration per method on EMDS-7.

Method	Backbone	Params (M)	FLOPs (G)	AP50	AP75	mAP	AR	Recall	Precision
ATSS	ResNet-50	32.21	172.82	69.2	59.3	52.1	65.2	93.3	79.0
Cascade R-CNN	ResNet-18	56.21	160.70	75.7	70.3	63.9	58.2	76.6	87.4
CenterNet	ResNet-18	19.19	130.70	75.6	69.4	59.5	59.5	83.3	84.1
Conditional DETR	ResNet-50	43.46	86.55	22.3	6.8	10.1	24.0	56.5	14.3
CornerNet	Hourglass-104	201.00	1501.98	65.8	62.2	58.4	60.5	77.7	73.9
DAB-DETR	ResNet-50	43.71	87.87	21.7	7.7	9.8	28.1	64.4	18.1
Deformable DETR	ResNet-50	40.11	166.82	47.6	36.7	32.8	59.3	90.3	49.8
DINO	ResNet-50	47.62	238.23	64.7	60.3	55.6	**76.8**	**96.8**	71.1
Faster R-CNN	ResNet-18	28.49	132.98	**77.5**	**73.0**	**64.0**	58.7	78.4	**89.9**
FCOS	ResNet-50	32.21	168.79	63.7	48.8	43.5	61.8	92.5	72.8
FoveaBox	ResNet-101	55.32	238.08	25.5	20.3	18.2	60.2	91.8	21.7
FreeAnchor	ResNeXt-101	55.80	256.55	35.9	31.8	28.1	67.2	93.1	35.2
FSAF	ResNeXt-101	94.23	371.46	29.2	23.8	21.7	62.3	92.6	25.9
GFL	ResNeXt-101	50.97	243.12	69.0	62.7	56.3	69.3	94.6	81.6
PAA	ResNet-50	32.21	172.82	66.1	55.3	49.5	66.3	93.8	75.1
RepPoints	ResNet-101	55.83	226.01	62.6	50.6	45.8	66.4	93.6	69.8
RetinaNet	ResNet-18	20.62	146.02	13.4	10.3	9.2	58.1	87.5	9.2
RTMDet-L	CSPNeXt	–	–	70.5	65.1	58.5	74.7	96.4	81.3
RTMDet-M	CSPNeXt	–	–	71.1	65.4	58.4	74.7	96.5	82.3
RTMDet-S	CSPNeXt	–	–	70.3	65.1	57.6	72.7	95.7	81.1
RTMDet-Tiny	CSPNeXt	–	–	66.0	60.2	52.5	72.4	96.5	75.4
RTMDet-X	CSPNeXt	–	–	73.5	68.4	60.9	74.1	96.4	84.8
Sparse R-CNN	ResNet-101	125.22	194.13	35.0	29.5	26.5	54.3	82.9	31.4
SSD	MobileNetV2	3.65	6.23	55.7	43.0	38.8	53.1	79.9	65.9
VFNet	ResNeXt-101	51.42	229.90	68.3	61.9	55.6	69.7	94.5	80.4
YOLOF	ResNet-50	43.28	84.06	59.9	41.7	38.2	43.8	77.4	63.6
YOLOv3	MobileNetV2	3.70	13.70	57.0	50.5	44.1	46.8	67.8	64.4
YOLOX-M	CSPDarknet	–	–	40.4	26.6	25.9	58.4	88.1	46.6
YOLOX-Tiny	CSPDarknet	–	–	2.1	0.4	0.9	12.5	27.4	0.0
YOLOX-X	CSPDarknet	–	–	32.0	19.5	18.6	45.5	74.3	29.1

For each detector, we report the best-performing backbone setting in terms of mAP, together with efficiency and accuracy metrics. Params denotes the number of model parameters (in millions), and FLOPs denotes the computational complexity (in GFLOPs). Accuracy-related metrics are scaled by 100 for readability. AP50 and AP75 denote average precision at IoU thresholds 0.50 and 0.75, respectively; mAP is the COCO-style average over IoU = 0.50:0.05:0.95; AR denotes average recall. Bold values indicate the best results within each column.

We also report **Average Recall (AR)** to reflect the detector's ability to retrieve true instances under the same IoU evaluation protocol. Specifically, AR is computed by averaging recall over the same set of IoU thresholds *T*:


$$AR_{0.50:0.05:0.95}=\frac{1}{\left|T\right|}\underset{t\in T}{\sum }\text{Recall}\left(t\right).$$

For completeness, Table 1 additionally includes overall **Recall** and **Precision** values (computed from the final matched predictions under the evaluation protocol) to provide an intuitive view of the trade-off between missed detections and false positives.

## 4 Experiment

### 4.1 Implementation details

All experiments are implemented with the PyTorch framework and the MMDetection toolbox, and are trained under a unified hardware environment using an NVIDIA GeForce RTX 4090 GPU to reduce confounding effects from computational resources. For reproducibility, we use a fixed random seed across all runs (covering randomness in model initialization and data loading), and we set the number of training epochs to 50 for every method.

Regarding optimization, different detectors may prefer different optimizers and learning-rate scheduling strategies. Therefore, we follow a method-compatible configuration for the optimizer type and the decay schedule, using either Stochastic Gradient Descent (SGD) or AdamW depending on the detector, while keeping the initial learning rate fixed at 0.02 for all methods. This design balances fairness, reproducibility, and practical compatibility: it avoids penalizing a detector with an ill-suited optimizer or schedule, while still constraining key experimental factors for more interpretable cross-method comparisons. At the same time, we acknowledge that different detectors may have different method-specific optimal recipes. Therefore, the resulting benchmark should be interpreted as a controlled relative comparison under a common protocol, rather than as a per-method performance maximization study. To complement the accuracy-oriented evaluation, we also report model efficiency indicators, including the number of parameters and FLOPs, for the best configuration of each detector. FLOPs are computed under the same input setting used in the benchmark to provide a consistent measure of computational complexity across methods. These statistics are intended to support a more practical comparison of accuracy–efficiency trade-offs for potential deployment scenarios.

In addition, since the evaluated methods adopt different backbone architectures, we do not use any pre-trained weights for model initialization. All models are trained from scratch to eliminate potential bias introduced by external pre-training data, objectives, or initialization advantages, so that performance differences primarily reflect detector design and learning mechanisms.

During inference, each detector is evaluated under its standard test-time configuration to preserve method-consistent prediction filtering and post-processing. The values reported in Table 1 are computed accordingly. Specifically, recall is evaluated at an IoU threshold of 0.50 with a maximum of 1,000 detections per image, while precision is evaluated at an IoU threshold of 0.75 at the operating point corresponding to a recall level of 0.5.

### 4.2 Methods evaluated

We evaluate a broad set of object detectors that represent diverse technical paradigms, including proposal-based two-stage models, dense one-stage detectors (anchor-based and anchor-free), keypoint-based formulations, and Transformer-based end-to-end approaches:

- **SSD (Liu et al., 2016)** adopts a classical one-stage design that performs dense classification and box regression on multiple feature scales.
- **RetinaNet (Lin et al., 2017b)** is an anchor-based one-stage detector that introduces *focal loss* to mitigate severe foreground–background imbalance.
- **Faster R-CNN (Ren et al., 2016)** follows a canonical two-stage pipeline that generates region proposals and refines them via region-wise classification and regression.
- **Cascade R-CNN (Cai and Vasconcelos, 2018)** cascades multiple regression stages to progressively improve localization under stricter IoU thresholds.
- **Sparse R-CNN (Sun et al., 2021)** replaces dense proposal generation with a learnable set of sparse proposals that are iteratively refined.
- **ATSS (Zhang et al., 2020)** assigns positive samples adaptively for each instance based on statistical characteristics, reducing hand-crafted assignment heuristics.
- **PAA (Kim and Lee, 2020)** formulates sample assignment in a probabilistic manner to select more reliable positives and stabilize optimization.
- **FSAF (Zhu et al., 2019)** enables feature-level selection across the pyramid so that each instance can be supervised on the most suitable feature level.
- **FreeAnchor (Zhang X. et al., 2019)** relaxes heuristic anchor matching by casting assignment into an optimization objective to improve matching quality.
- **FoveaBox (Kong et al., 2020)** defines positives within a central region to reduce ambiguous assignments near object boundaries.
- **FCOS (Tian et al., 2019)** is an anchor-free detector that predicts classification scores and regresses distances from a location to the four box sides.
- **RepPoints (Yang et al., 2019)** represents objects using a deformable point set and converts it to bounding boxes for flexible geometric modeling.
- **GFL (Li et al., 2020)** couples classification confidence with localization quality to improve the ranking consistency of high-quality predictions.
- **VFNet (Zhang et al., 2021)** explicitly models IoU-aware localization quality to emphasize learning and selecting high-quality boxes.
- **CenterNet (Zhou et al., 2019)** follows a center-based formulation that detects object centers and regresses size/offset to form bounding boxes.
- **CornerNet (Law and Deng, 2018)** is a keypoint-based detector that predicts paired corners and groups them to generate bounding boxes.
- **YOLOv3 (Redmon and Farhadi, 2018)** represents the real-time one-stage family with end-to-end dense prediction as the core design principle.
- **YOLOF (Chen et al., 2021)** advocates an efficient design that relies on a simplified feature setting while maintaining competitive performance.
- **YOLOX (Ge et al., 2021)** improves the YOLO pipeline with refinements such as decoupled heads for better accuracy and stability.
- **RTMDet (Lyu et al., 2022)** is a recent high-performance and efficient one-stage detector that provides a strong accuracy–speed trade-off.
- **DETR (Carion et al., 2020)** formulates detection as set prediction and trains end-to-end via bipartite matching between predictions and ground truth.
- **Conditional DETR (Meng et al., 2021)** introduces conditional mechanisms in attention to improve optimization and accelerate convergence.
- **Deformable DETR (Zhu et al., 2020)** adopts deformable attention to improve efficiency and strengthen multi-scale modeling, which is often beneficial for small objects.
- **DAB-DETR (Liu S. et al., 2022)** parameterizes queries as dynamic anchor boxes to facilitate localization learning.
- **DINO (Zhang et al., 2022)** further improves DETR-style training and matching, typically achieving better convergence and accuracy.

### 4.3 Results

Table 1 reports the best configuration for each detector on EMDS-7 under the unified benchmark protocol. For readability, all metrics are scaled by 100 (i.e., percentage form). Overall, EMDS-7 remains challenging: while several methods achieve high AP50, the COCO-style *mAP* averaged over IoU 0.50:0.05:0.95 is notably lower, reflecting the difficulty of precise localization in microscopy images.

Among all evaluated detectors, **Faster R-CNN** achieves the best overall accuracy with *mAP* = 64.0, and also attains the highest **AP50** (**77.5**) and **AP75** (**73.0**). **Cascade R-CNN** follows closely with *mAP* = 63.9, suggesting that proposal-based two-stage pipelines remain highly effective for environmental microorganism detection. On the one-stage side, **RTMDet-X** performs competitively (*mAP* = 60.9), while **CenterNet** (*mAP* = 59.5) and **CornerNet** (*mAP* = 58.4) also provide strong results. Quality-aware dense detectors such as **GFL** (*mAP* = 56.3) and **VFNet** (*mAP* = 55.6) offer solid baselines, whereas earlier one-stage designs (e.g., **SSD**, **YOLOF**) are relatively weaker in terms of *mAP*.

The table also includes **Recall** and **Precision** to provide an intuitive view of missed detections versus false positives. **Faster R-CNN** yields the highest **Precision** (**89.9**) with moderate recall (78.4), indicating strong selectivity and a low false-positive tendency. In contrast, **DINO** attains the highest **Recall** (**96.8**) and **AR** (**76.8**), but with lower precision (71.1), suggesting a more aggressive retrieval behavior that favors finding more instances at the cost of increased false positives. This trade-off is particularly relevant for microscopy scenarios where sensitivity for counting and tolerance to false alarms may differ by application.

When comparing paradigms, proposal-based detectors (Faster R-CNN and Cascade R-CNN) deliver the strongest *mAP*, consistent with their region-wise refinement and robust localization. Modern one-stage detectors substantially narrow the gap: RTMDet variants achieve *mAP* between 52.5 and 60.9, demonstrating that efficient dense detectors can be highly competitive under the same input normalization and training budget. Transformer-based detectors exhibit mixed behavior: **DINO** reaches *mAP* = 55.6 with very high recall, while earlier DETR variants such as **Conditional DETR** and **DAB-DETR** obtain much lower *mAP* (around 10), indicating that end-to-end set prediction can be sensitive to optimization and may require additional tailoring for this small-object, cluttered microscopy setting.

In summary, EMDS-7 favors detectors that can maintain both high detection sensitivity and robust localization. Two-stage detectors provide the best overall accuracy, while recent one-stage designs (e.g., RTMDet-X) offer strong performance with a favorable simplicity/efficiency profile. The precision–recall and AR trends further highlight that different paradigms may prioritize different operating points, motivating future work on improving high-IoU localization and reducing false positives under complex microscopic backgrounds.

## 5 Analysis

This chapter analyzes the benchmark results reported in Table 1. Overall, proposal-based two-stage detectors achieve the strongest overall performance on EMDS-7 in terms of COCO-style *mAP* under the benchmark setting used in this study (all values are reported in percentage form). **Faster R-CNN** achieves the best overall accuracy (*mAP* = 64.0) and also leads at AP50 = 77.5 and AP75 = 73.0, indicating strong classification and localization quality under both moderate and stricter IoU requirements. **Cascade R-CNN** is a close runner-up (*mAP* = 63.9), suggesting that, under the present benchmark setting, multi-stage refinement is advantageous for environmental microorganism detection on EMDS-7.

Modern one-stage detectors narrow the gap substantially. **RTMDet-X** reaches *mAP* = 60.9 and maintains high AP50 and AP75, suggesting that recent dense detectors can remain competitive when sample assignment and localization-quality modeling are sufficiently improved. This is particularly important for EMDS-7, where small instances and cluttered backgrounds require the detector to preserve discriminative multi-scale features while suppressing false positives. Keypoint/center-based methods also perform strongly (**CenterNet** *mAP* = 59.5, **CornerNet** *mAP* = 58.4), implying that alternative object parameterizations can be effective when structural cues are informative enough for localization.

The Precision/Recall and AR columns highlight different operating characteristics. **Faster R-CNN** yields the highest **Precision** (89.9), reflecting a conservative decision boundary with fewer false positives. In contrast, **DINO** attains the highest **Recall** (96.8) and **AR** (76.8), indicating stronger instance retrieval but comparatively more false positives. This trade-off is important in downstream monitoring scenarios, where some applications prioritize sensitivity (counting) while others prioritize reliability (low false-alarm rate). Finally, Transformer-based detectors show mixed behavior: the stronger variant (**DINO**) is competitive, whereas earlier DETR variants (e.g., Conditional DETR and DAB-DETR) are markedly weaker in this setting. This gap suggests that Transformer-based detection on EMDS-7 is highly sensitive to optimization quality and multi-scale representation, especially under from-scratch training and in the presence of small objects with ambiguous boundaries.

### 5.1 AP degradation across IoU thresholds

Table 2 further reports AP across IoU thresholds from 0.50 to 0.95 for the best configuration of each method (values in %). A consistent trend across detectors is the monotonic decrease of AP as IoU becomes stricter, confirming that precise localization is a major difficulty in environmental microorganism microscopy. This is expected due to small object sizes, ambiguous boundaries, and background clutter. In EMDS-7, many objects occupy only a small fraction of the image, so even minor coordinate deviations can lead to substantial IoU reduction. Therefore, the drop from moderate to strict IoU thresholds reflects not only general localization difficulty, but also the particular sensitivity of small-object detection to boundary errors.

**Table 2:** Best configuration per method on EMDS-7 with AP across IoU thresholds (%).

Method	Backbone	AP50	AP55	AP60	AP65	AP70	AP75	AP80	AP85	AP90	AP95
ATSS	ResNet-50	69.2	68.6	67.2	65.9	62.8	59.3	52.2	44.4	25.1	6.4
Cascade R-CNN	ResNet-18	75.7	75.5	74.7	74.0	72.4	70.3	65.8	**61.7**	49.8	18.9
CenterNet	ResNet-18	75.6	75.5	74.8	74.0	72.6	69.4	62.9	53.5	29.3	7.3
Conditional DETR	ResNet-50	22.3	20.4	18.2	14.9	11.0	6.8	4.6	2.0	0.5	0.1
CornerNet	Hourglass-104	65.8	65.0	64.3	63.9	63.4	62.2	59.3	57.6	**51.0**	**31.6**
DAB-DETR	ResNet-50	21.7	19.6	18.0	14.0	10.4	7.7	3.8	1.8	0.6	0.2
Deformable DETR	ResNet-50	47.6	45.8	44.3	42.2	40.3	36.7	30.3	24.5	15.1	1.6
DINO	ResNet-50	64.7	63.6	63.4	62.9	61.6	60.3	57.0	54.3	46.2	22.5
Faster R-CNN	ResNet-18	**77.5**	**77.1**	**77.0**	**76.5**	**74.5**	**73.0**	**67.8**	59.4	43.6	13.5
FCOS	ResNet-50	63.7	62.1	60.2	58.0	54.8	48.8	39.7	31.1	13.9	2.5
FoveaBox	ResNet-101	25.5	24.9	24.1	23.6	22.7	20.3	17.9	13.2	8.3	1.9
FreeAnchor	ResNeXt-101	35.9	35.4	34.9	34.4	33.4	31.8	28.8	24.9	16.5	4.7
FSAF	ResNeXt-101	29.2	28.7	27.9	26.9	25.4	23.8	21.6	17.2	12.5	3.7
GFL	ResNeXt-101	69.0	68.7	68.5	67.5	65.9	62.7	58.9	51.7	38.2	11.9
PAA	ResNet-50	66.1	64.6	64.1	62.5	58.9	55.3	51.1	41.0	25.9	5.3
RepPoints	ResNet-101	62.6	61.7	60.0	58.0	54.8	50.6	45.9	38.5	21.7	4.0
RetinaNet	ResNet-18	13.4	12.9	12.5	12.1	11.2	10.3	8.5	6.1	3.7	1.1
RTMDet-L	CSPNeXt	70.5	70.3	70.1	69.0	67.5	65.1	61.5	53.9	41.9	15.0
RTMDet-M	CSPNeXt	71.1	70.9	70.5	68.4	66.9	65.4	61.7	53.9	40.8	14.2
RTMDet-S	CSPNeXt	70.3	70.0	69.4	68.3	67.0	65.1	60.4	50.9	39.1	15.1
RTMDet-Tiny	CSPNeXt	66.0	65.2	64.9	64.4	62.9	60.2	54.0	47.8	33.7	6.3
RTMDet-X	CSPNeXt	73.5	73.3	73.1	72.5	70.9	68.4	63.2	56.7	41.8	15.9
Sparse R-CNN	ResNet-101	35.0	34.6	34.2	33.0	31.5	29.5	26.5	21.5	15.7	3.2
SSD	MobileNetV2	55.7	54.5	52.5	50.7	47.5	43.0	38.1	27.7	15.5	2.6
VFNet	ResNeXt-101	68.3	67.8	67.4	66.7	65.6	61.9	57.8	51.5	36.7	12.8
YOLOF	ResNet-50	59.9	58.6	57.5	54.7	48.6	41.7	32.7	19.3	8.3	1.1
YOLOv3	MobileNetV2	57.0	57.0	56.5	56.0	54.4	50.5	46.5	36.7	21.4	5.1
YOLOX-M	CSPDarknet	40.4	39.9	37.8	36.4	32.0	26.6	21.6	16.1	7.8	0.3
YOLOX-Tiny	CSPDarknet	2.1	2.0	1.6	1.4	0.5	0.4	0.3	0.1	0.1	0.0
YOLOX-X	CSPDarknet	32.0	30.9	29.4	27.0	24.5	19.5	13.3	6.7	2.2	0.0

Comparing methods, **Faster R-CNN** maintains the strongest AP over a wide IoU range (leading from AP50 through AP80), indicating robust localization stability rather than merely high AP50. **Cascade R-CNN** shows particularly strong performance at moderately high IoU (e.g., AP85), consistent with the benefit of cascaded refinement for improving box quality. Notably, **CornerNet** achieves the best results at very strict thresholds (AP90 and AP95), suggesting that its keypoint-based formulation can produce more accurate boxes for a subset of instances, even if its overall *mAP* is slightly below the top two-stage baselines.

Among end-to-end Transformer detectors, **DINO** exhibits relatively smooth degradation and remains competitive at higher IoU thresholds (e.g., AP85–AP95), aligning with its strong AR/Recall behavior in Table 1. In contrast, weaker DETR variants degrade rapidly and stay low across all thresholds, implying that improved matching/optimization and multi-scale modeling are critical for Transformers in this domain.

Overall, the AP-sweep analysis suggests that, under the current experimental setting, future improvements on EMDS-7 may benefit from emphasizing high-IoU localization (e.g., better box regression for small instances and boundary ambiguity) and false-positive suppression under complex backgrounds, since gains at strict IoU thresholds are more directly reflected in demanding localization criteria.

### 5.2 Backbone analysis

**Figure 1:**
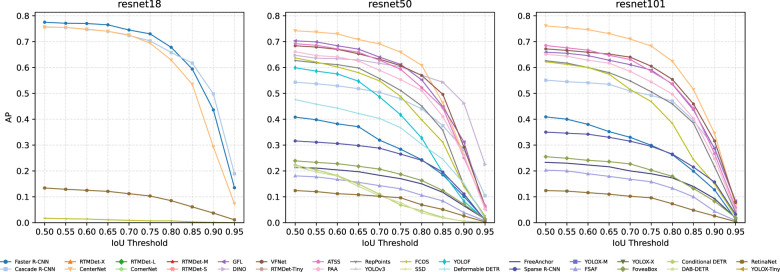
AP versus IoU threshold on EMDS-7 for detectors instantiated with different ResNet backbones. **Left to right**: ResNet-18, ResNet-50, and ResNet-101. Each curve shows the method's AP at IoU thresholds from 0.50 to 0.95 (step 0.05); colors/markers are consistent across subplots to facilitate comparison. The overall downward trends highlight the increasing difficulty of precise localization at higher IoU, while the relative separations between curves indicate how backbone capacity interacts with different detection paradigms.

Several detectors in our benchmark can be instantiated with different backbone capacities. To examine how backbone choice affects localization behavior on EMDS-7, we compare AP–IoU curves under ResNet-18, ResNet-50, and ResNet-101 backbones. Figure 1 overlays the AP degradation trajectories across IoU thresholds for all evaluated methods under each backbone, enabling a direct cross-backbone comparison of both accuracy level and stability as the matching criterion becomes stricter.

From the figure, deeper backbones do not yield a uniform improvement across methods when training from scratch under the same protocol. For strong two-stage baselines, competitive performance is already achieved with a lightweight backbone, and increasing depth does not consistently translate into better high-IoU AP. This suggests that, for microscopy images dominated by small objects and ambiguous boundaries, localization errors are not determined by backbone representation power alone, but also by how effectively the detector head and training process convert features into accurate box regression. As a result, simply increasing depth does not guarantee better high-IoU performance. Therefore, the limited gain from deeper backbones in this benchmark should be interpreted as the result of interactions among dataset scale, training regime, detector design, and localization difficulty, rather than as evidence that backbone depth is generally unimportant.

In contrast, a subset of dense detectors shows clearer sensitivity to backbone capacity, where stronger backbones tend to maintain higher AP in the mid-to-high IoU range. This indicates that richer feature hierarchies can benefit methods that rely on dense prediction and fine-grained feature discrimination, although the gains remain bounded as IoU approaches 0.90–0.95.

A consistent phenomenon across all three backbones is the sharp performance drop at very strict IoU thresholds, confirming that high-precision localization remains challenging regardless of backbone depth. Therefore, improving EMDS-7 performance may require targeted localization enhancements (e.g., small-object oriented feature design, better box regression objectives, or boundary-aware supervision) rather than simply scaling backbone capacity.

### 5.3 Recall analysis

**Figure 2:**
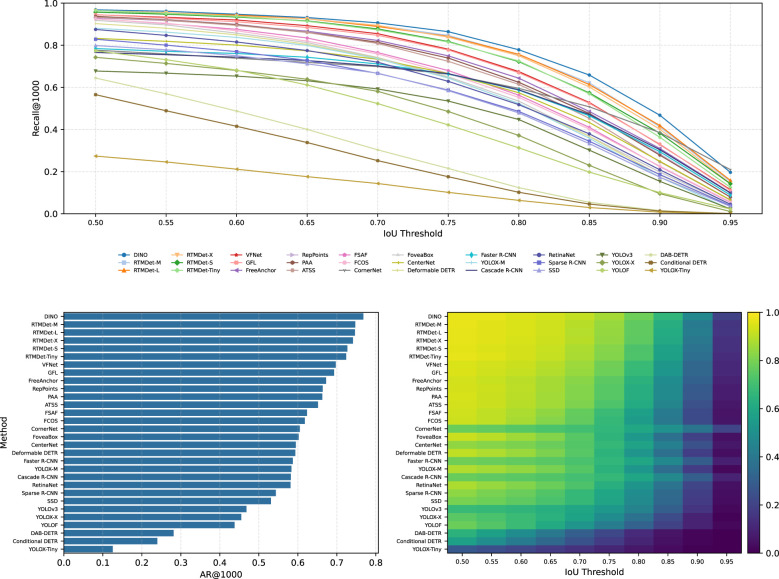
Recall analysis on EMDS-7. **Top:** Recall@1000 versus IoU threshold (0.50–0.95, step 0.05). **Bottom–left:** AR@1000 ranking across methods. **Bottom–right:** heatmap view of Recall@1000 across IoU thresholds. Higher curves/bars indicate stronger instance retrieval, while steeper drops at high IoU reflect increased sensitivity to localization errors.

To further characterize detection behavior beyond AP, we analyze recall under different IoU thresholds. Figure 2 summarizes **Recall@1000** as a function of IoU and the corresponding **AR@1000** (average recall over IoU 0.50:0.05:0.95) across all evaluated methods. This view highlights how aggressively each detector retrieves instances and how quickly recall deteriorates as localization requirements become stricter.

Overall, most methods exhibit a monotonic decline in recall as IoU increases, confirming that tight localization is difficult in microscopy images with small instances and ambiguous boundaries. Among all detectors, **DINO** achieves the strongest retrieval behavior, showing the highest Recall@1000 across most IoU thresholds and the top AR@1000. Modern dense detectors (especially the **RTMDet** family) also maintain consistently high recall, indicating strong sensitivity for finding instances under moderate localization constraints.

In contrast, several methods demonstrate noticeably lower recall, and their recall curves drop sharply as IoU approaches 0.90–0.95. This suggests that these detectors either miss a larger portion of instances (lower sensitivity) or struggle to produce sufficiently accurate boxes to satisfy strict matching. Importantly, high recall does not necessarily imply the best overall accuracy. Methods with very high recall may retrieve more true instances, but may also assign positive confidence to ambiguous regions in cluttered backgrounds, which reduces precision. Conversely, more conservative detectors may suppress false positives more effectively, but at the cost of missing weak or hard-to-localize instances. Therefore, the recall-based analysis complements AP by revealing whether a detector tends to favor sensitivity or selectivity under the EMDS-7 benchmark.

## 6 Conclusion

In this paper, we established a reproducible benchmark for environmental microorganism object detection on the EMDS-7 dataset. Under a unified protocol (fixed data split, consistent input normalization, and from-scratch training without external pre-training), we conducted a systematic comparison across diverse detector families, including two-stage proposal-based methods, modern one-stage dense detectors, keypoint-based formulations, and Transformer-based end-to-end approaches. In addition to reporting COCO-style *mAP* over IoU 0.50:0.05:0.95, we analyzed performance trends across IoU thresholds, backbone choices, and recall behavior to provide a more complete diagnostic view of detector characteristics in microscopy imagery.

Our results show that, under the unified benchmark setting adopted in this study, proposal-based two-stage detectors achieve the strongest overall accuracy on EMDS-7, with Faster R-CNN and Cascade R-CNN performing best among the evaluated methods. Meanwhile, recent one-stage detectors (e.g., RTMDet variants) substantially narrow the gap under the same training budget. The AP-sweep analysis reveals a consistent degradation as IoU becomes stricter, indicating that precise localization is a key bottleneck, likely driven by the small object sizes, ambiguous boundaries, and cluttered backgrounds typical of environmental microorganism microscopy. Backbone experiments further suggest that, under the current protocol, simply increasing backbone depth does not necessarily lead to improved high-IoU localization, highlighting the importance of detector-head design and optimization stability. Finally, recall-based analysis shows that different paradigms operate at different points on the sensitivity–selectivity spectrum: some favor higher recall at the cost of more false positives, while others prioritize precision through more conservative retrieval.

Overall, the benchmark results and diagnostic analyses provide a useful reference for future research on environmental microorganism detection. Promising directions include improving high-IoU localization for small instances, developing boundary-aware or noise-robust supervision, and reducing false positives induced by complex microscopic backgrounds. We hope this benchmark can support more reproducible and targeted research under controlled settings, while the conclusions drawn here should be interpreted within the scope of the present study.

## Data Availability

The datasets presented in this study can be found in online repositories. The names of the repository/repositories and accession number(s) can be found in the article/supplementary material.
